# Best practices for bioinformatic characterization of neoantigens for clinical utility

**DOI:** 10.1186/s13073-019-0666-2

**Published:** 2019-08-28

**Authors:** Megan M. Richters, Huiming Xia, Katie M. Campbell, William E. Gillanders, Obi L. Griffith, Malachi Griffith

**Affiliations:** 1 Division of Oncology, Department of Internal Medicine, Washington University School of Medicine, St. Louis, MO 63110 USA; 2 McDonnell Genome Institute, Forest Park Avenue, Washington University School of Medicine, St. Louis, MO 63108 USA; 3 Division of Hematology and Oncology, Medical Plaza Driveway, Department of Medicine, University of California, Los Angeles, Los Angeles, CA 90024 USA; 4 Department of Surgery, South Euclid Avenue, Washington University School of Medicine, St. Louis, MO 63110 USA; 5 Siteman Cancer Center, Parkview Place, Washington University School of Medicine, St. Louis, MO 63110 USA; 6 Department of Genetics, South Euclid Avenue, Washington University School of Medicine, St. Louis, MO 63110 USA

## Abstract

Neoantigens are newly formed peptides created from somatic mutations that are capable of inducing tumor-specific T cell recognition. Recently, researchers and clinicians have leveraged next generation sequencing technologies to identify neoantigens and to create personalized immunotherapies for cancer treatment. To create a personalized cancer vaccine, neoantigens must be computationally predicted from matched tumor–normal sequencing data, and then ranked according to their predicted capability in stimulating a T cell response. This candidate neoantigen prediction process involves multiple steps, including somatic mutation identification, HLA typing, peptide processing, and peptide-MHC binding prediction. The general workflow has been utilized for many preclinical and clinical trials, but there is no current consensus approach and few established best practices. In this article, we review recent discoveries, summarize the available computational tools, and provide analysis considerations for each step, including neoantigen prediction, prioritization, delivery, and validation methods. In addition to reviewing the current state of neoantigen analysis, we provide practical guidance, specific recommendations, and extensive discussion of critical concepts and points of confusion in the practice of neoantigen characterization for clinical use. Finally, we outline necessary areas of development, including the need to improve HLA class II typing accuracy, to expand software support for diverse neoantigen sources, and to incorporate clinical response data to improve neoantigen prediction algorithms. The ultimate goal of neoantigen characterization workflows is to create personalized vaccines that improve patient outcomes in diverse cancer types.

## Background

The adaptive immune system has inherent antitumor properties that are capable of inducing tumor-specific cell death [1, 2]. CD8+ and CD4+ T cells, two immune cell types that are critical to this process, recognize antigens bound by class I and II major histocompatibility complexes (MHC) on the cell surface, respectively. After antigen recognition, T cells have the ability to signal growth arrest and cell death to tumor cells displaying the antigen, and also release paracrine signals to propagate an antitumor response. Neoantigens are specifically defined here as peptides derived from somatic mutations that provide an avenue for tumor-specific immune cell recognition and that are important targets for cancer immunotherapies [3–5]. Studies have shown that, in addition to tumor mutational burden (TMB), high neoantigen burden can be a predictor of response to immune checkpoint blockade (ICB) therapy [6, 7]. This treatment strategy targets the signaling pathways that suppress antitumor immune responses, allowing the activation of neoantigen-specific T cells and promoting immune-mediated tumor cell death. Therefore, accurate neoantigen prediction is vital for the success of personalized vaccines and for the prioritization of candidates underlying the mechanism of response to ICB. These approaches have great therapeutic potential because neoantigen-specific T cells should not be susceptible to central tolerance.

**Fig. 1:**
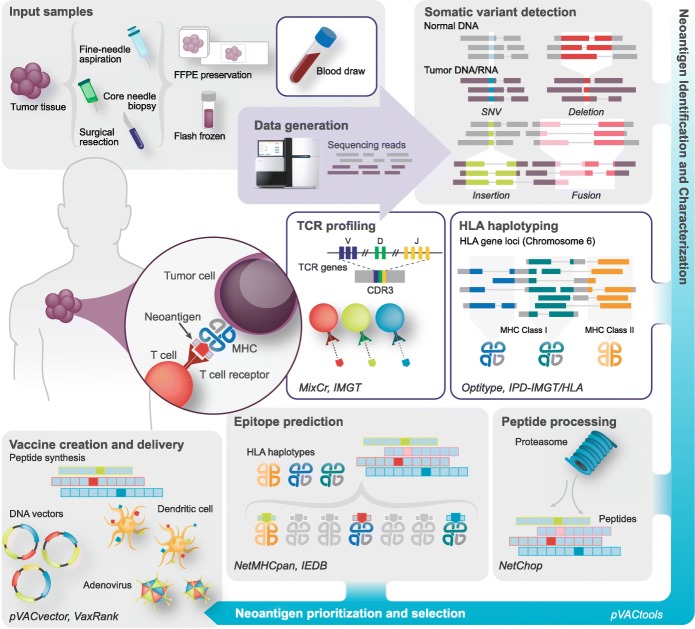
Overview of the bioinformatic characterization of neoantigens. Major analysis steps in a comprehensive workflow for neoantigen characterization are depicted in a simplified form. For each component, critical concepts and analysis considerations are indicated. Specific exemplar bioinformatics tools for each step are indicated in *italics*. Starting at the *top left*, patient sequences are analyzed to determine human leukocyte antigen (HLA) types and to predict the corresponding major histocompatibility complexes (MHC) for each tumor. Somatic variants of various types, including single nucleotide variants (SNVs; *blue*), deletions (*red*), insertions (*green*), and fusions (*pink*), are detected and the corresponding peptide sequences are analyzed with respect to their predicted expression, processing, and ability to bind the patient’s MHC complexes. Candidates are then selected for vaccine design and additional analyses are performed to assess the T cell response. *Abbreviations*: *CDR3* complementarity-determining region 3, *FFPE* formalin-fixed paraffin-embedded, *IEDB* Immune Epitope Database, *TCR* T cell receptor

With the advent of next generation sequencing (NGS), researchers can now rapidly sequence a patient’s DNA and RNA before analyzing these sequencing data to predict neoantigens computationally. This process requires several steps, each involving the use of bioinformatics tools and complex analytical pipelines (Fig. 1; Table 1). Matched tumor–normal DNA sequencing data are processed and analyzed to call somatic mutations of various types. Human leukocyte antigen (HLA) haplotyping is performed to determine a patient’s HLA alleles and the corresponding MHC complexes. Finally, RNA sequencing (RNA-seq) data are used to quantify gene and transcript expression, and can verify variant expression prior to neoantigen prediction. Multiple pipelines exist to identify candidate neoantigens that have high binding affinities to MHC class I or II. Additional steps are subsequently required to prioritize them for clinical use in personalized vaccines and to address manufacturing and delivery issues [8, 9].

**Table 1:** Tool categories, a brief description of their roles and a list of exemplar tools

Tool categories	Function and examples
Alignment	DNA: Bwa-mem [161] RNA: STAR [162], HISAT2 [163]
Sequence data QC	Picard (http://broadinstitute.github.io/picard/), FastQC (https://github.com/s-andrews/FastQC), RSeQC [164], MultiQC (https://github.com/ewels/MultiQC) (note that MultiQC supports an extensive list of additional QC tools)
Variant callers	SNV/Indel: Mutect [19], Strelka [20], VarScan2 [21], SomaticSniper [22], Shimmer [165], VarDict [166], deepSNV [167], EBCall [40] Structural variants: Pindel [43], Manta [168], Lumpy [169] Fusions: STAR-Fusion [48], Pizzly [47], SOAPfuse [170], JAFFA [49], ChimPipe [171], GFusion [50], INTEGRATE [51]
Variant call format (VCF) manipulation	Vt decompose (https://github.com/atks/vt), GATK (https://github.com/broadinstitute/gatk) (e.g., SelectVariants, CombineVariants, LeftAlignAndTrimVariants)
Variant annotation	Variant Effect Predictor (VEP) (https://github.com/Ensembl/ensembl-vep) (SNV/Indel), AGFusion [172] (RNA fusions), bam-readcount (https://github.com/genome/bam-readcount), VAtools (https://github.com/griffithlab/VAtools)
Gene or transcript abundance estimation	StringTie [173], Kallisto [174]
HLA typing	Class I: Optitype [69], Polysolver [70] Class I and II: Athlates [70, 175], HLAreporter [176], HLAminer [176, 177], HLAscan [72, 178], HLA-VBSeq [72], PHLAT [71], seq2HLA [73], xHLA [74]
Peptide processing	Proteasome cleavage: NetChop20S [89], NetChopCterm [89], ProteaSMM [89, 90], PAProC [179] (Class I), PepCleaveCD4 [91] (Class II) TAP transport efficiency: [90] (no specific tool name)
MHC binding predictors	Class I predictors: SMM [111], SMMPMBEC [112], Pickpocket [113], NetMHC [114], NetMHCpan [87], NetMHCcons [180], MHCflurry [102], MHCnuggets [181], MHCSeqNet [103], EDGE [104] Class II predictors: SMMAlign [111], NNAlign [182], ProPred [183], NetMHCII(2.3) and NetMHCIIpan(3.2) [116], TEPITOPE [184], TEPITOPEpan [185], RANKPEP [186], MultiRTA [187], OWA-PSSM [188]
Neoantigen prioritization pipelines	pVACtools [8], Vaxrank [9], MuPeXI [119], TIminer [120], Neoepiscope [189], TSNAD [190], EpiToolKit [123], NeoepitopePred [122], TepiTool (IEDB) [191], ScanNeo [192], CloudNeo [193], NeoPredPipe [118]
Peptide creation and delivery	pVACtools [8] (pVACvector), Vaxrank [9] (manufacturability)
TCR repertoire profiling	LymAnalyzer [194], MiXCR [147], MIGEC [148], pRESTO [195], TRUST [196], TraCeR [145], VDJtools [197], VDJviz [198], ImmunoSEQ [199], GLIPH [151]
Immune cell profiling	CIBERSORT [152], TIMER [153], quanTIseq [200], immunophenogram [201], MCPcounter [202], SSGSEA [203]

This table compiles the current state of tools, databases, and other resources that are used in neoantigen pipelines. Although many of the steps that are outlined may involve the integration of multiple tools for comparable predictions (e.g., using multiple somatic variant callers or MHC-binding-affinity predictors), this table summarizes more options than are needed in a single workflow. For an example of the specific combination of tools, parameter settings, and order of operations used in a real end-to-end workflow that is based on our own practices, please refer to our online tutorial for precision medicine bioinformatics (https://pmbio.org/). *TAP* Transporter associated with antigen processing

The general concept of neoantigens and their role in personalized immunotherapies have been extensively reviewed elsewhere [10–12]. Although experimental methods exist to assess neoantigens (e.g., mass spectrometry (MS)), the focus of this review is a comprehensive survey of computational approaches (tools, databases, and pipelines) for neoantigen characterization. The ultimate goal is to discover neoepitopes, the part of the neoantigen that is recognized and bound by T cells, but current workflows are largely focused on predicting MHC-binding antigens with limited prediction of recognition by T cells or therapeutic potential. We have been particularly inspired by the use of computational approaches in human clinical trials involving personalized neoantigen vaccines alone or in combination with ICB. A rapid expansion of the number and diversity of these trials has occurred over the past few years, but there is limited community consensus on approaches for neoantigen characterization. Adoption of standards for the accurate identification of neoantigens and for the reporting of their features will be critical for the interpretation of results from early-stage trials and for the optimization of future trials. This review is focused on human clinical data; nevertheless, neoantigen characterization work involving model organisms (such as mice) will be critical to advance the field, and many of the tools and approaches described herein may be applied to these model systems with appropriate modifications. In addition to describing emerging best practices, we highlight the current limitations and critical areas for the improvement of the computational approaches needed to understand the immunogenicity of neoantigens.

## Neoantigen identification

Two types of antigens that can induce an antitumor response are tumor-specific antigens (or neoantigens) and tumor-associated antigens (TAA). Neoantigens contain altered amino-acid sequences that result from non-silent somatic mutations, whereas TAAs, which may originate from endogenous proteins or retroviruses, are selectively expressed or overexpressed by tumor cells but may also be expressed by non-tumor cell populations [13]. This review focuses on the detection and selection of neoantigens, but many analytical steps that are used can apply to other antigen types. Considerations such as sample type (fresh frozen, formalin-fixed paraffin-embedded (FFPE) tissue or circulating tumor DNA (ctDNA)), tumor type (solid or blood), biopsy site, and sequencing approach (DNA, RNA, or targeted sequencing) can impact somatic variant detection and interpretation, and should be taken into account during data processing and downstream analysis [13–16]. In addition, tumors that exhibit high intratumoral heterogeneity can require alternative methods, such as collecting multiple biopsies per tumor [17].

Somatic variant callers identify single nucleotide variants (SNVs) from tumor and matched non-tumor DNA sequence data, such as whole genome, or more commonly, whole exome sequencing (WES) data [18]. Three common limitations to SNV calling—low frequency variant detection, distinguishing germline variants from tumor in normal contamination, and removing sequencing artifacts—have been addressed by the variant callers discussed below. MuTect2 [19] and Strelka [20] have high sensitivity in detecting SNVs at low allele fractions, enabling accurate subclonal variant detection. VarScan2 [21] and SomaticSniper [22] require higher allele fractions for recognizing variants but can improve performance in cases of tumor in normal contamination [23, 24]. MuTect2 can further exclude sequencing or alignment artifacts by implementing a panel-of-normals file, containing false positives detected across normal samples. Running multiple variant calling algorithms simultaneously is recommended and can result in higher detection accuracy. For example, Callari et al. [25] achieved 17.1% higher sensitivity without increasing the false-positive rate by intersecting a single variant caller’s results from multiple alignment pipelines and then combining the intersected results from two callers, MuTect2 and Strelka, to achieve a final consensus. The list of variant callers mentioned here is not exhaustive (see Table 1 for additional options) and high-quality pipelines using different combinations are certainly possible. Regardless of the combination of callers used, manual review of matched tumor–normal samples in Integrative Genomics Viewer (IGV) [26], with a documented standard operating procedure, is recommended to further reduce false positives [27]. In addition to IGV, targeted sequencing approaches such as custom capture reagents can be utilized for further variant validation.

Recently, neoantigen vaccine trials for melanoma demonstrated that SNV-derived neoantigens can expand T cell populations [28] and induce disease regression [29, 30]. However, recent studies have also increased appreciation for diverse neoantigen sources beyond simple SNVs, including short insertions and deletions (indels) [31], fusions [32, 33], intron retentions [34], non-coding expressed regions [35], exon–exon junction epitopes [36], B cell receptor (BCR) and T cell receptor (TCR) sequences for B and T cell malignancies, respectively [37], and more [38].

Frameshift mutations resulting from insertions and deletions create alternative open reading frames (ORFs) with novel tumor-specific sequences that are completely distinct from those that encode wild-type antigens. A pan-cancer analysis of 19 cancer types from The Cancer Genome Atlas demonstrated that frameshift-derived neoantigens were present in every cancer type [31]. This mutation type also occurs frequently in microsatellite instability high (MSI-H) colon and other cancers and correlates with higher CD8+ T cell infiltrate in the tumors [31, 39]. For calling indels, in addition to Strelka, EBCall [40] demonstrates the least sensitivity to coverage variability [41, 42]. Pindel [43] specializes in calling larger indels, from 0.50–10 kilobases in length, and structural variants. Though these are popular indel callers, they are only a subset of the available tools (see Table 1 for additional options).

Translocations may result in tumor-specific fusion genes, which can alter the reading frame and provide novel junction sequences. Researchers recently investigated the presence of translocations in osteosarcoma, characterized by high genomic instability [44], and discovered multiple fusion-derived junction-spanning neoantigens [45]. The identification of novel sequences resulting from inter- and intrachromosomal rearrangements in mesothelioma also resulted in the prediction of multiple neoantigens for each patient [46]. Many tools have been developed to predict fusion genes from RNA-seq and/or whole genome sequencing (WGS) data; recent tools include pizzly [47], STAR-fusion [48], JAFFA [49], GFusion [50], and INTEGRATE [51] (refer to Table 1). The main limitation of these fusion callers is the low level of overlap between tools; they largely achieve high sensitivity at the cost of low specificity. The presence of many false positives makes accurate detection difficult, but this can be mitigated by using multiple tools [52] and by requiring predictions to be supported by multiple callers and/or data types (e.g., WGS and RNA-seq).

In addition to mutation-derived neoantigens from known protein-coding genes, noncoding regions have immunogenic potential. Noncoding transcripts can be created from noncoding exons, introns, and untranslated regions (UTRs), as well as from non-canonical reading frames in the coding region [53]. Laumont et al. [35] investigated traditionally noncoding sequences using liquid chromatography tandem-MS (LC-MS/MS) and RNA sequencing (RNA-seq) in leukemia and lung cancer patients and found an abundance of antigens, both mutated and unmutated, from noncoding regions.

Recent publications have shown that aberrant tumor-specific splicing patterns can create neoantigens. Smart et al. [54] found an approximately 70% increase in total predicted neoantigens after including retained intron sequences along with SNVs in the prediction pipeline. Novel junctions created by exon skipping events, or neojunctions, have been shown to create neoantigens [36]. Tumor-specific splicing patterns can also cause distinct alternative 3′ or 5′ splice sites, known as splice-site-creating mutations, and these mutations are predicted to create an average of 2.0–2.5 neoantigens per mutation [55].

In addition to the neoantigen sources discussed above, many alternative sources can create neoantigens. For example, V(D) J recombination and somatic hypermutation generate immunoglobulin (Ig) variable region diversity in B and T lymphocytes, and the resulting unique receptor sequences can function as neoantigens in heme malignancies [37, 56]. Further, researchers have demonstrated that peptides with post translational modifications, including phosphorylation and O-GlcNAcylation, in primary leukemia samples can serve as MHC-I restricted neoantigens [57, 58]. Alternative translation events resulting from non-AUG start codons and viral sequences that are associated with tumors (e.g., human papilloma virus (HPV)) are also a source of neoantigens [59–63]. Overall, neoantigen identification requires a sensitive, accurate, and comprehensive somatic variant calling pipeline that is capable of robustly detecting all of the variant classes that are relevant for a tumor type (Table 2).

**Table 2:** Key analysis considerations and practical guidance for clinical neoantigen workflows

Analysis area	Guidance
Reference genome sequences	The choice of human reference genome sequences can have important implications for various analysis steps throughout neoantigen characterization workflows. A consistent build or assembly (e.g., GRCh38 or GRCh37) of the genome should be used throughout the analysis. Even if two resources provide annotations that are based on the same assembly, they may organize or name sequences differently and might follow different conventions for representing ambiguous or repetitive sequences. They may also drop some sequences (e.g., alternative contigs) or add sequences that are not part of the official assembly (e.g., ‘decoy’ sequences). The use of reference files from multiple sources for different tools is difficult to avoid but should be pursued cautiously. For example, the naming of chromosomes and contigs used for DNA read alignment and variant calling should be compatible (identical) to those used in transcript annotations. Otherwise, this may prevent correct prediction of the protein sequences of neoantigens
Use of alternative contigs in the reference genome	The inclusion or exclusion of alternative contigs from the latest human reference genome build can have important implications for HLA typing tools such as xHLA [74]. In particular, if a tool assumes that all relevant reads for HLA typing can be extracted from an existing alignment (rather than performing de novo re-alignment of all reads), it matters whether some of these reads may have been placed on alternative contigs for the HLA locus of chromosome 6. Some HLA typing approaches avoid this issue by aligning all reads directly to a database of known HLA gene sequences (e.g., from the IPD-IMGT/HLA resource). This has the disadvantage that without competitive alignment of each read to the whole genome, some reads may be misaligned to the known HLA sequences and this may affect accuracy during HLA typing. A reference genome alignment approach, in which the diversity of HLA loci is properly represented in the reference, avoids this concern and has the potential to leverage alignments that may have already been produced for variant calling. For example, all reads aligning to the HLA loci of chromosome 6, the corresponding alternative contigs (if present in the reference), and unaligned reads could be extracted from a BAM file and used for HLA typing
Transcript annotation build versions	Transcript annotation resources (e.g., Ensembl, RefSeq, GENCODE, and Havana) update their transcript sequences and associated annotations more frequently than new reference genome sequence builds/assemblies are released. For example, Ensembl is currently on version 96, the 21st update since the latest release of the human reference genome, build GRCh38. As with reference genome builds, it is highly desirable to use a consistent set of transcript annotations across the steps of a neoantigen characterization workflow. For example, the transcripts used to annotate somatic variants should be the same as those used to estimate transcript and gene abundance from RNA data
Variant detection sensitivity	Correct neoantigen identification and prioritization rely on somatic and germline variant detection (for proximal variant analysis) and variant expression analysis. QC analysis of both DNA and RNA data should be performed to assess the potential for a high false-negative rate in detecting somatic variants that might lead to neoantigens, to identify germline variants in phase with somatic variants that influence the peptide sequence bound by MHC, or to assess the expression of these variants. Tumor samples vary significantly in their level of purity and genetic heterogeneity. Common strategies to achieve high sensitivity in variant detection involve increasing the average sequencing depth and combining results from multiple variant callers
Combining variants from multiple callers	The majority of somatic variant callers now use the widely adopted variant call format (VCF). Furthermore, many toolkits now exist for the manipulation of these files, including merging. However, because of the complexity and flexibility of the VCF specification (https://samtools.github.io/hts-specs/VCFv4.2.pdf), the existence of multiple versions of the specification, and the varying interpretations of VCF rules observed in the output of somatic variant callers, great care must be taken when combining multiple VCFs and using these merged results. Important considerations include: (i) variant justification and parsimony such as left aligning or trimming variants to harmonize those that can be correctly represented at multiple positions without changing the resulting sequence (e.g., GATK LeftAlignAndTrimVariants); (ii) normalization of multi-allelic variants by separating multiple variant alleles that occur at a single position into multiple lines in a VCF (e.g., vt decompose); (iii) harmonization of sequence depths, allele depth, and allele fraction values that may be calculated inconsistently by different variant callers through the use of an independent counting tool, such as bam-readcount (https://github.com/genome/bam-readcount); (iv) determining the final status for each variant (PASS or filters failed; e.g., GATK SelectVariants); and (v) choosing the variant INFO and FORMAT fields to represent in the final merged VCF
Variant refinement (‘manual review’)	Somatic variant calling pipelines remain subject to high rates of false positives, particularly in cases of low tumor purities or of insufficient depth of sequencing of tumor (or matched normal) samples or sub-clones. Prior to final neoantigen selection, all somatic variants should be carefully reviewed for possible alignment artifacts, systematic sequencing errors, nearby in-phase proximal variants, and other issues using a standard operating procedure for variant refinement, such as that outlined by Barnell et al. [27]
Choosing RNA and DNA variant allele fraction (VAF) cutoffs	It is impossible to define universal VAF recommendations because of the varying distribution of VAFs observed for tumor samples with different sequencing depths, tumor purity/cellularity, genetic heterogeneity, and degree of aneuploidy. The interpretation of each individual candidate may be influenced by one or more of these factors. In general, however, neoantigens corresponding to somatic variants with higher VAFs (in both DNA and RNA) will be considered with higher priority. Estimating the overall purity of the DNA sample by VAF distribution and distinguishing founding clones from sub-clones requires accurate assignment of each variant to a copy number estimate. Accepting or rejecting candidates on the basis of VAF requires a nuanced approach that takes the characteristic of each tumor into account. For example, a variant with a relatively low DNA VAF may be accepted in some cases if sequencing depth at the variant position was marginal, leading to a less accurate VAF estimate. A variant with a relatively high DNA VAF may be rejected if RNA-seq analysis shows strong evidence of allele-specific expression (of the wild-type allele)
Interpretations that depend on RNA quality assessment	Attempting to define expressed and unexpressed variants by RNA-seq analysis is a common feature of many neoantigen characterization workflows. Applying hard filters in this area should be pursued with great caution. All interpretation of RNA-seq should be accompanied by comprehensive QC analysis of the data [204]. A lack of evidence for expression in RNA-seq data may not be definitive evidence of non-expression of a variant because not all genes can be robustly profiled by RNA-seq (for example, very small genes may be poorly detected by standard RNA-seq libraries [205]). Tumor samples that are obtained in clinical workflows, particularly those involving FFPE, may frequently result in poor-quality RNA samples. In these cases, the requirements for expression support may be relaxed when nominating neoantigen candidates. Furthermore, some variants occur within a region of a gene that is difficult to align reads to. In these cases, robust apparent expression of the gene may still be used to nominate a neoantigen even in the absence of evidence supporting the expression of the variant allele itself. Use of spike-in control reagents and routine profiling of reference samples can be helpful in determining consistent expression value cutoffs (e.g., FPKM or TPM values) across samples. In the absence of reliable gene or variant expression readout for an individual tumor, robust expression of the gene in tumors of the same type may be used to prioritize neoantigens
Assessing variant clonality	A major consideration in the interpretation of DNA VAFs of variants is the assessment of tumor clonality. Neoantigens corresponding to variants that reside in the founding clone are inherently more valuable therapeutically than those residing in tumor sub-clones, because the former have the potential to target the elimination of all tumor cells. In personalized cancer vaccine designs, after correcting for ploidy and tumor purity, VAFs should be interpreted to prioritize neoantigens that correspond to founding clones
Variant types and agretopicity	Calculation of ‘agretopicity’ (also known as ‘differential agretopicity index’ [121], or ‘wild-type/mutant binding affinity fold change’) refers to an attempt to estimate the degree to which a neoantigen’s ability to bind to MHC differs from that of its corresponding wild-type sequence. This calculation thus depends on the ability to define a wild-type counterpart for each neoantigen sequence. For non-synonymous SNVs, the wild-type counterpart sequence is assumed to be a peptide of the same length without the amino acid substitution. For many other variant types, defining a counterpart wild-type sequence is much less obvious because the variant may lead to a sequence that is entirely novel and shares little or no homology with the wild-type sequences encoded from the region of the variant. These include frameshift mutations caused by deletions or insertions, translocations that lead to in-frame or frame-shifted RNA fusions, alternative isoforms caused by aberrant RNA splicing that lead to partial or complete intron retention, novel exon junctions, and so on. In these cases, agretopicity values are typically not calculated and may be reported as not applicable. This should be taken into consideration when prioritizing variants of mixed type using these values. Interpretation of agretopicity is primarily relevant when the mutant amino acid(s) involve anchor residues of the MHC [206]
HLA naming conventions	Neoantigen characterization workflows should consistently adopt the widely used standards and definitions for the communication of histocompatibility typing information [207]. Briefly, HLA alleles are named using an HLA prefix followed by a hyphen, gene designation, asterix separator, and four fields of digits delimited by colons (e.g., HLA-A*02:101:01:02 N). The four fields (typically of two or three digits each) represent the allele group, specific HLA protein, synonymous changes in the coding region, and non-coding differences, respectively. Several popular HLA typing bioinformatics tools only report two field HLA types. The first two fields are generally sufficient for pMHC binding affinity predictions because they describe any polymorphisms that influence the protein sequence of MHC. However, three-field typing might be desirable for patient-specific assessment of expression, because even silent variations in the DNA sequence of the HLA locus may influence read assignments to specific alleles
HLA typing (class I vs II typing)	Accurate HLA typing is critical to neoantigen characterization workflows. Without accurate knowledge of the HLA alleles of an individual, it is not possible to predict pMHC binding and presentation on tumor cells or cross presentation by APCs. Many clinical- or research-grade HLA typing assays are available, and they rely on PCR amplification or, more recently, NGS data. HLA typing results from a CAP/CLIA-regulated assay are expected to be robust and remain the gold standard. In addition to clinical HLA typing, there are now several bioinformatics tools and pipelines available for HLA typing from whole genome, exome, or RNA-seq data (Table 1). Several groups have now conducted comparisons between the results of these tools and clinical assay results and have reported high concordance, particularly for class I typing. Class II typing remains challenging, with fewer tools available and poorer consistency between the results of these tools and clinical assays. Use of clinical-typing results remains advisable for class II. As in other areas of neoantigen analysis, the use of a consensus approach involving multiple tools has become a common strategy to increase confidence in HLA typing results [208]
HLA typing (selection of data type and samples)	Several options are available for input data when performing HLA typing from NGS data, including DNA (WES or WGS) or RNA-seq data. RNA-seq data often exhibit highly variable coverage across the HLA loci, potentially leading to variable accuracy in typing for each. Coverage data from exome data may vary depending on the exome reagent’s design (probes selected against HLA regions) and capture efficiency. Care should be taken to evaluate sufficient read coverage for each HLA locus when assessing HLA-typing confidence. WGS data may exhibit comprehensive breadth of coverage, but generally at the expense of overall depth of coverage (again coverage achieved for the HLA loci specifically should be evaluated). In addition to data type, there is also the choice of whether to perform HLA typing using data from the tumor itself or a reference normal sample. The normal sample has the advantage that it should represent the germline HLA alleles present in both the initiating cells of the tumor and the antigen presenting cells of the immune system (relevant for cross-presentation). In many clinical and research workflows, the quality of genomic DNA may be higher in the normal sample than in the tumor (often a FFPE-preserved sample). The genomic DNA of the tumor may also be complicated by aneuploidy that affects the HLA loci (which is important to observe and has the potential to interfere with HLA typing). HLA typing using the tumor DNA data has the advantage that it may more accurately reflect the MHC binding and presentation of neoantigens on the surface of the targeted tumor cells. However, it is important to note that HLA-typing tools are, for the most part, not designed for de novo HLA typing; instead, they seek to determine which of a list of known alleles best explain the sequence reads of a given data set. HLA-typing tools also generally do a poor job of reporting HLA-typing confidence. At present, identification of the loss of expression or a somatic mutation of an HLA allele in a tumor is perhaps best treated as a separate exercise from HLA typing. One strategy for choice of data for HLA typing is to use all of the datasets available (DNA and RNA, normal and tumor), to note any discrepancies, and to investigate them
HLA expression and mutation	Loss of expression of MHC molecules by HLA deletion (or downregulation) and somatic mutation of HLA loci have both been identified as possible resistance mechanisms for immunotherapies [76]. It is therefore desirable for neoantigen characterization workflows to incorporate examination of HLA expression and somatic mutation in the tumor. Unfortunately, very few tools and best practices exist for these examinations. Given the sequence diversity of the HLA loci across individuals, when estimating the expression of HLA transcripts in a tumor, it is desirable to customize the reference transcripts used (e.g., from the IPD-IMGT/HLA resource) for each individual’s HLA type by using the results of HLA genotyping to select the matching transcript sequences (three-field matched) for expression abundance estimation (for example, with Kallisto)
Class I versus class II allele specification for binding prediction algorithms	Class I HLA alleles are typically supplied to binding affinity prediction algorithms using a standard two-field format (e.g., HLA-A*02:01). However, class II alleles are often supplied as a pair using valid two-field pairing combinations (e.g., DQA1*01:01-DQB1*06:02) to reflect the functional dimers of class II MHC. Peptide MHC prediction tools will typically document the syntax and list the valid pairings for which binding-affinity predictions are supported
Proximal variation	Neoantigen selection pipelines often focus entirely on one variant or position at a time, and consider it to be independent of all nearby variations. It is important to examine candidates carefully to determine whether nearby variation exists that is both in phase (on the same allele) and close enough to influence the peptide sequence and therefore the MHC binding predictions [117]
Peptide-length considerations	Many human class I pMHC binding affinity prediction tools support a range of peptide lengths for each individual HLA allele (e.g., IEDB supports lengths of 8–14 amino acids for class I for HLA-A*01:01). Typically, although multiple lengths are supported, the peptides that are found to have strong binding will be highly biased towards the lengths actually favored by the allele (for example, many human HLA alleles strongly favor nonamers). The open binding groove of MHC class II is thought to support a greater range of peptide lengths. This is reflected in some class II binding prediction tools, although it should be noted that the IEDB API and web resource currently enforce a length of 15 amino acids only
Relationship between genomic variants and short peptides	There is a complex relationship between genomic variants and the short peptide neoantigen candidates that they might represent. Though rare, it is possible for multiple distinct somatic variations to result in the same amino acid change (for example, several single nucleotide substitutions affecting a single triplet codon) and therefore they might lead to identical neoantigens. If these variations were to occur on opposite alleles, it might be important to analyze them separately because they could differ in expression level and/or their proximal variants, giving rise to distinct peptides. Other ways in which a single genomic variant can give rise to distinct short peptides for pMHC binding prediction include: (i) a homozygous somatic variant representing two distinct alleles; if these alleles are in phase with one or more nearby heterozygous proximal variants, distinct peptide sequences may result; (ii) SNVs expressed in different RNA transcripts or isoforms that differ in their reading frame at the position of the variant, in the inclusion or exclusion of nearby alternative exons, or in the nearby use of alternative RNA splicing donor or acceptor sites; and (iii) multiple short peptides that result simply from shifting the ‘register’ of the somatic variant in a short sequence or from the use of multiple peptide lengths (e.g., 8–11-mers) during the prediction of pMHC binding affinity. In some ways, mostly similar peptide sequences do not matter in peptide vaccine design because a longer peptide will ultimately incorporate several of them into a single peptide sequence. However, pMHC binding prediction algorithms require that you supply a short sequence, of a specific length with the variant in a particular register, and each of these lead to different predicted binding affinity values. Making decisions about how to summarize, collapse, filter, and select representatives is one of the complexities that are addressed by pipelines such as pVACtools
Importance of transcript annotation quality and choice to select a single transcript variant annotation	Peptides that are considered as potential neoantigens are generally derived from the anticipated open reading frame of a known or predicted transcript sequence. A common consideration in variant effect annotation is whether to allow annotations for each variant against multiple transcripts or whether a single representative transcript should be selected. If choosing a single transcript for each gene, multiple strategies exist including the following: (i) use of a pre-selected automatically determined or manually curated choice of ‘canonical’ transcript for each gene; or (ii) considering all transcripts but selecting the single transcript that results in the most confident and/or consequential predicted functional impact. The latter is the basic intent of the ‘--pick’ option of the Ensembl Variant Effect Predictor (VEP), which chooses one block of annotations for each variant using an ordered set of criteria (refer to the VEP documentation for extensive details). The benefit of choosing a single transcript for the annotation of each variant is simplicity, and in many cases, it will result in the selection of a suitable peptide sequence for neoantigen analysis. However, the downside is that distinct peptides may not be considered and the peptide corresponding to the selected annotation is not guaranteed to be the best. Note that a single variant may be assigned annotations for: multiple genes, multiple transcripts of the same gene, and multiple effects for the same transcripts. For example, a single variant can be annotated as splicing-relevant (near the edge of an exon causing exon skipping) and also as missense (causing a single amino acid substitution). The same variant could be silent for a different transcript of the same gene and have a regulatory impact on a transcript of another gene. Making sensible automated choices about how to choose and report neoantigen candidates that correspond to these variants is a complexity that neoantigen characterization workflows seek to address
Importance of transcript annotation quality	When using VEP, it can be important to consider the Transcript Support Level assigned by Ensembl. As described above, this classification is one of many factors that are considered in choosing a single ‘best’ transcript for the annotation of variants. Occasionally, a variant annotation will be reported with a dramatic effect (e.g., nonsense) but on further inspection, it is found that this effect is only true for a transcript that is poorly supported by sequence evidence, and another more reliable transcript would lead to different candidate neoantigen sequences
Selection of pMHC binding affinity prediction cutoff(s)	Many pMHC binding prediction tools report binding strength as an IC50 value in nanomolar (nM) units. Peptides that have a binding affinity of less than 500 nM are commonly selected as putative strong binding peptides. However, the widespread use of this common binding strength metric may provide a false sense of consistency. Trusting a simple cutoff of 500 nM from a single algorithm should be avoided, but combining scores from multiple algorithms should also be pursued very cautiously. The range, median, and even shape of distribution of IC50 scores varies dramatically across algorithms, even when applied to exactly the same peptides [8]. Further complicating the selection process, the accuracy of the IC50 estimates varies across HLA alleles (reflecting the biased and variable strength of experimental evidence used to train generalized predictive models). Partially addressing this concern, the IEDB now provides recommended ‘per allele’ binding-score thresholds for the selection of strong binders
Interpretation of binding affinity from multiple binding prediction algorithms	Given the variability in IC50 predictions across binding prediction algorithms, some neoantigen workflows involve the use of multiple binding prediction tools and attempt to calculate or infer a consensus. Best practices for determining such a consensus are poorly articulated, and limited gold-standard independent validation data sets exist to evaluate the accuracy of divergent predictions. Unsophisticated but pragmatic approaches currently involve reporting the best score observed, calculating the median score, determining average rank values, or manually visualizing the range of predictions across algorithms for promising candidates, before making a qualitative assessment
Neoantigen candidate reporting, visualization, and final prioritization	Prior to the final review of candidates, the automated filtering of variants and peptides that do not meet basic criteria (VAFs, binding affinity, and so on) is performed to provide a more interpretable result. As discussed above, a single genomic variant can lead to many candidate peptide sequences (resulting from alternative reading frames, peptide lengths, registers, and so on). At the time of final candidate review and selection, a common strategy is to use a pipeline that will automatically choose a single representative (best) peptide for each variant in a filtered result. Similarly, a condensed report may be generated to present only the most important information about each candidate. Final assessment of a candidate neoantigen can easily involve the consideration of 20–50 specific data fields. Review of this data in spreadsheet form can be time-consuming and inefficient, and can make it difficult to consider some data in the context of a cohort of comparators (for example, expression values are often best interpreted relative to reference samples). Tools such as pVACviz are now emerging to facilitate more efficient visual interfaces for neoantigen candidate review
Vaccine manufacturing strategy	In the case of personalized cancer vaccine trials, the method of vaccine delivery can influence bioinformatics tool selection and other analysis considerations. For example, if candidates are to be encoded in a DNA vector, a tool such as pVACvector may be used to determine the optimal ordering of the peptide candidates. Owing to the combinatorial nature of candidate peptide sequence ordering, and the need to examine all pairs for junctional epitopes, this is currently one of the most computationally expensive and time-consuming steps of these workflows. Similarly, if peptides are to be synthesized for a peptide vaccine, there is a need to predict possible problems with synthesizing each peptide (for example, by calculating ‘manufacturability’ scores)

A detailed summary of analysis and interpretation best practices and nuances that should be considered when implementing a neoantigen identification workflow. Topics are covered in an order that corresponds to the flow of major steps discussed in the main body and depicted in Fig. 1. For further nuanced details on how to put the following guidance into practice, please refer to our tutorial on precision medicine bioinformatics (https://pmbio.org/). *Abbreviations*: *CAP* College of American Pathologists, *CLIA* The Clinical Laboratory Improvement Amendments, *FPKM* fragments per kilobase of exon model per million reads mapped, *TPM* transcripts per million

## HLA typing, expression, and mutation analysis

T cell priming depends in part on neoantigen presentation on the surface of dendritic cells, a type of professional antigen presenting cells (APCs). Dendritic cells engulf extracellular proteins, process the peptides, and present the neoantigens on MHC I or II molecules. MHC in humans is encoded by the HLA gene complex, which is located on chromosome 6p21.3. This locus is highly polymorphic, with over 12,000 established alleles and more in discovery [64]. Because HLA genes are extensively individualized, precise HLA haplotyping is essential for accurate neoantigen prediction. The gold standard for this process is clinical HLA typing using sequence-specific PCR amplification [65]. More recently, NGS platforms such as Illumina MiSeq and PacBio RSII have been combined with PCR amplification to sequence the HLA locus [66]. However, clinical typing can be laborious and expensive, so a common alternative approach is computational HLA typing using the patient’s WGS, WES, and/or RNA-seq datasets, which are typically created from a peripheral blood sample, except in heme malignancies, where a skin sample is often used (Table 2).

HLA class I typing algorithms (Table 1) have reached up to 99% prediction accuracy when compared to curated clinical typing results [67, 68]. Although many class I typing algorithms exist, OptiType [69], Polysolver [70], and PHLAT [71] currently have the highest reported accuracies [67, 68, 70]. Despite the high precision of class I tools, class II HLA typing algorithms remain less reliable and require additional development to improve their prediction accuracy. Few benchmarking studies that consider class II algorithm accuracy have been performed, but a combined class I and II comparison demonstrated that PHLAT [71], HLA-VBSeq [72], and seq2HLA [73] performed well with WES and RNA-seq data [67]. Additional HLA typing algorithms, xHLA [74] and HLA-HD [75], have recently been published and show comparable accuracies to those of the tools described above.

Tumor-specific T cell recognition relies on efficient antigen presentation by tumor cells, so one mechanism of resistance to immunotherapies is the loss or attenuated expression of the HLA gene loci. Recently, researchers have identified transcriptional HLA repression in a patient with Merkel cell carcinoma (MCC) following treatment with autologous T cell therapy and ICB [76]. The authors found that the transcriptional silencing can be reversed in ex vivo cultures by treatment with 5-aza and other hypomethylating agents, indicating that reversing the epigenetic silencing of the HLA genes could sensitize tumors that exhibit HLA downregulation in response to immunotherapies [77].

Genetic changes at the HLA locus can be determined by Polysolver [70], an algorithm that detects HLA-specific somatic mutations from computational HLA typing and variant calling of the tumor HLA locus. Somatic mutation analysis of head and neck squamous cell carcinoma (HNSCC), lung cancer, and gastric adenocarcinoma cohorts demonstrated that HLA mutations are prevalent in all three cancer types [78–80]. In addition, HLA mutations (particularly frameshifts, nonsense, and splicing mutations) are enriched towards the beginning of the genes or within functional domains, where they would be expected to result in a loss-of-function phenotype [70]. Another tool, LOHHLA, can identify copy number variations in the HLA locus that result in loss of heterozygosity [81].

Additional components of the antigen presenting machinery, including B2M and TAP (Transporter associated with antigen processing), have been shown to accrue mutations and to exhibit altered expression patterns in tumors. In lung cancer and MSI-CRC, mutations or biallelic loss of *B2M* causes lack of class I HLA presentation [82, 83]. Downregulation of *B2M*, *TAP1*, and *TAP2* expression has also been shown to inhibit tumor antigen presentation [84, 85] and correlate with metastatic breast cancer phenotypes [86]. Identifying and characterizing altered HLA and associated presentation genes will allow clinicians to prioritize neoantigens that bind to expressed and unmutated alleles.

## Predicting peptide processing

Recognition of a peptide-MHC (pMHC) complex by the T cell is a complex process with many steps and requirements. Most of the attention in the field has been focused on predicting the binding affinity between the patient’s MHC molecule and a given peptide sequence, as this is believed to provide much of the specificity of the overall recognition [87]. However, even if a peptide has strong MHC binding prediction, the prediction may be meaningless if upstream processing prevents the actual loading of that peptide. In general, pipelines generate k-mer peptides using a sliding window that is applied to the mutant protein sequence, and these peptide sequences are subsequently fed into algorithms that predict the affinity of the peptide to the corresponding MHC. However, not all of the k-mers can be generated in vivo due to the limitations of the immune proteasome. In addition, only a subset of generated peptides will be transported into the appropriate cellular compartments and will interact with MHC molecules. These aspects of peptide processing, specifically immune proteasome processing and peptide cleavage, must be considered and several tools have been developed to address this component specifically [88].

For both the MHC class I and II pathways, an important upstream step prior to pMHC interaction is proteolysis, which refers to the degradation of proteins into peptides, particularly by the immunoproteasome. Multiple tools are now available to capture the specificity of proteasomes and to predict the cleavage sites that are targeted by different proteases. These tools include NetChop20S [89], NetChopCterm [89], and ProteaSMM [89, 90] for MHC class I antigens, and the more recent PepCleaveCD4 and MHC NP II for MHC class II antigens [91, 92]. Algorithms that have been developed in this area are generally trained on two different types of data, in vitro proteasome digestion data or in vivo MHC-I and -II ligand elution data. The neural network-based prediction method NetChop-3.0 Cterm has been shown to have the best performance in predicting in vivo proteolysis that provides peptide sources for MHC class I antigen presentation [88]. Cleavage site predictions for MHC class II epitopes show promise, but have yet to be validated for predicting immunogenicity [88, 92].

For MHC class I antigen processing, peptide fragments are generated from proteins that are present in the cytoplasm and transported by the TAP protein into the endoplasmic reticulum (ER), where the peptide is loaded onto an MHC molecule. Thus, in addition to tools focusing on the process of proteolysis, other tools have also been developed to predict the efficiency of peptide transportation on the basis of affinity to TAP proteins. Different methods have been employed in an attempt to determine which peptides have high affinity for TAP binding, including simple/cascade support vector machine (SVM) models [93, 94] and weight matrix models [95]. To address the entirety of this process, the Immune Epitope Database (IEDB) has also developed a predictor for the combination of these processes (proteasomal cleavage/TAP transport/MHC class I) [90, 96].

In the MHC class II pathway, the peptides are mostly exogenous and enter the endosome of APCs through endocytosis. As endosomes mature into late endosomal compartments, acidity levels increase and serine, aspartic, and cysteine proteases are activated. Proteins, upon exposure to a series of proteases, are degraded into potential antigens for presentation. MHC class II molecules are assembled in the ER and transported to these high acidity late endosomes, also known as MHC-II compartments (MIIC). Here, peptides can bind to class II molecules and are protected from destructive processing [97, 98]. In contrast to the protein denaturation in the MHC class I processing pathway, cleavage in the MHC class II pathway occurs on folded proteins. Predictors for class II peptide preprocessing prior to MHC binding show the important role that secondary structures play in such reactions, as multiple measures related to secondary structures were found to be highly correlated with the predicted cleavage score [91]. Consideration of secondary structure will be critical to the future development of tools predicting class II processed peptides. However, although the class I antigen processing pathway has been studied extensively, researchers have only recently started to focus on class-II-specific neoantigens as promising results have been shown in cancer immunotherapies [99–101]. There remains a great need to develop supporting tools and algorithms to characterize class-II-specific neoantigens.

For the purposes of neoantigen prioritization, it is important to take into account processing steps such as peptide cleavage and TAP transport when using binding prediction algorithms that were trained on in vitro binding data. Recently, published binding prediction algorithms have been transitioning to training on data generated in vivo, in which case the processing steps are accounted for intrinsically.

## MHC binding prediction

Neoantigen characterization pipelines have been established specifically to predict the binding of neoantigens to the patient’s specific class I and II MHC molecules (based on HLA typing). Algorithmic development and the refinement of reference data sets are very active in this area. Here, we describe the current state of the art with respect to algorithmic innovation and refinement of the major classes of data that are used to train these algorithms (largely from in vitro binding assays involving specific MHCs and peptide libraries or from MS-based approaches) [87, 102–104].

Peptides bind MHC molecules at a membrane-distal groove that is formed by two antiparallel α-helices overlaying an eight-strand β-sheet [97]. The peptide-binding region of the MHC protein is encoded by exons 2 and 3 of the corresponding *HLA* gene [105]. High allelic polymorphism allows the binding pocket of MHC molecules to recognize a range of different peptides sequences, and the positions that are involved in anchoring the peptide to the MHC molecule in particular vary for each HLA allele. The algorithms and training datasets for predicting pMHC binding remain an active area of development. Various methods have been employed in an attempt to capture the characteristics of peptide and MHC molecules that have a high probability of binding (Table 1).

Early algorithms have mostly focused on training using in vitro pMHC binding affinity measurement datasets. MHC peptide binding is thought to be the most selective step in the antigen presentation process, but sole consideration of peptide binding predictions still results in high rates of false-positive predictions of neoantigens for applications in personalized immunotherapy [28, 29]. This insufficiency probably results from the influence of other factors including the preprocessing of peptides, the stability of the pMHC complex [106, 107], and peptide immunogenicity [108]. Recently published MHC binding algorithms use either only peptidome data, generated from in vivo immunoprecipitation of pMHC complexes followed by MS characterization, or an integration of MS and binding-affinity data [87, 102, 104]. By directly examining ligands that are eluted from pMHC complexes identified in vivo, predictive models can capture features unique to peptides that have undergone the entire processing pathway. Over 150 HLA alleles have corresponding binding-affinity datasets available in IEDB (with highly variable amounts of data for each allele) [96]. By contrast, MS peptidome datasets are available for only approximately 55 HLA alleles [87], probably because of the lack of high-throughput characterization assays. However, continuous development in MS profiling techniques [109] may soon close the gap between the two types of data. Zhao and Sher [110] recently performed systematic benchmarking for 12 of the most popular pMHC class I binding predictors, with NetMHCpan4 and MHCflurry determined to have the highest accuracy in binding versus non-binding classifications. The analysis also revealed that the incorporation of peptide elution data from MS experiments has indeed improved the accuracy of recent predictors when evaluated using high-quality naturally presented peptides [110].

Different types of algorithmic approaches have been used to model and make predictions for the binding affinity of MHC class I molecules. Initially, predictors relied on linear regression algorithms and more specifically on stabilized matrix methods, such as SMM [111], SMMPMBEC [112], and Pickpocket [113]. However, recently published or updated predictors almost exclusively employ variations of neural networks [87, 102, 104, 114], as shown in Table 3. Linear regression assumes a linear contribution of individual residues to the overall binding affinity; however, while artificial neural networks require more training data, they are able to capture the nonlinear relationship between the peptide sequence and the binding affinity for the corresponding MHC molecules through hidden layers in their network architecture. Given the growing number of available training datasets, applications of artificial neural networks have been able to achieve higher accuracy than that provided by linear regression predictive methods [110].

**Table 3:** MHC class I binding algorithm comparison

Features/ software	Algorithm type used	Type of data used for training	Number of HLA alleles used for training	HLA alleles and peptide length that can be predicted	Output information
Pickpocket (2009)	Position-specific weight matrices	In vitro quantitative binding data (> 150,000 data points)	More than 150 different MHC molecules	HLA-A, −B, −C, −E and -G alleles, also for non-human primates, mice, cattle and pigs. Peptides of 8–12 in length	Prediction values are given in nM IC50 values
NetMHCcons (2012)	Integration of NetMHC 3.4, NetMHCpan 2.8 and PickPocket 1.1	In vitro binding affinity data	NetMHC 3.4 (94 MHC class I alleles), NetMHCpan 2.8 (> 120 different MHC molecules), PickPocket 1.1 (94 different MHC alleles)	Can predict peptides to any MHC molecule of known sequence. Peptides of 8–15 amino acids in length	Prediction values are given in nM IC50 values and as %rank to a set of 200,000 random natural peptides
NetMHC 4.0 (2016)	Artificial neural networks	In vitro binding affinity data	81 different human MHC alleles (HLA-A, −B, −C, and -E) and 41 animal alleles	81 different human MHC alleles (HLA-A, −B, −C, and -E) and 41 animal alleles. Any length but recommends 9 and discourages above 11 amino acids	Core position for binding within the peptide, interaction core sequence, affinity in nM, rank of prediction compared with 400,000 random natural peptides (strong binders %rank < 0.5), and so on
NetMHCpan 4.0 (2017)	Artificial neural networks	Binding affinity (> 180,000 data points) and eluted ligand (MS) data	172 human and other animal MHC molecules	Can predict peptides to any MHC molecule of known sequence	Core position for binding within peptide, interaction core sequence, affinity in nM, rank of the predicted affinity compared to a set of random natural peptides (strong binders %rank < 0.5), and so on
MHCnuggets (2017)	Gated recurrent neural networks	IC50 values from immuno-fluorescent binding experiments for pMHC Class I pairs (137,654 data points)	106 unique MHC alleles	Any MHC alleles, more reliable for alleles that are present in IEDB. Any peptide length is valid	IC50 binding affinity prediction
MHCflurry (2018)	Allele-specific feed forward neural networks	Binding affinity and eluted ligand (MS) data (> 230,735 data points)	Across 130 alleles from IEDB combined with benchmark dataset from Kim et al. [209]	112 alleles showed performance sufficient for their inclusion in predictor. Peptide lengths of 8–15 are supported	Affinity given in nM, percentile predictions across the models, and quantile of affinity prediction among large number of random peptides tested
EDGE (2019)	Deep neural network	Peptide sequences from HLA immunoprecipitation followed by MS characterization	Not explicitly specified	53 HLA alleles, 8–15-mer (inclusive)	Not explicitly specified

A direct comparison of a subset of popular MHC class I binding predictors showing their variability in algorithmic structure, training data, supported HLA alleles and valid peptide lengths

While prediction algorithms for MHC class I molecules are well developed, algorithms for MHC class II are fewer, less recently developed, and trained with smaller datasets. Unlike MHC class I molecules, class II molecules are heterodimeric glycoproteins that include an ɑ-chain and a β-chain; thus, MHC II molecules are more variable than MHC I molecules as a result of the dimerization of highly polymorphic alpha and beta chains. The binding pocket for class II molecules is open on both ends, which allows a larger range of peptides to bind. The most frequently observed lengths of peptides that bind to class II MHCs are between 13 and 25 amino acids [115], whereas those for class I typically fall between 8 and 15 amino acids [87]. Nevertheless, for any one particular MHC allele, the preferred number of amino acids may be much more constrained to one or two lengths. Algorithms built for class II predictions generally rely on matrix-based methods and ensembles of artificial networks. A selection of popular MHC class II binding prediction algorithms are summarized in Table 1 [116].

There is an extensive list of MHC binding prediction tools for both class I and class II molecules, but there remains a need not only to expand the training data for a larger range of HLA alleles but also to refine the type of training data being used in these algorithms. Although in vivo MS data capture the features of peptides that are naturally presented by MHC molecules, they cannot confirm whether such peptides are able to induce an immune response. Algorithms should ideally incorporate experimentally and clinically validated immunogenic peptides in their training and validation datasets. As ongoing neoantigen clinical trials produce more of such data, tool development and refinement in this area will also become possible.

## Neoantigen prioritization and vaccine design pipelines

Owing to the numerous factors that are involved in the process of antigen generation, processing, binding, and recognition, a number of bioinformatic pipelines have emerged with the goal of assembling the available tools in order to streamline the neoantigen identification process for different clinical purposes (such as predicting the response to ICB, designing peptide- or vector-based vaccines, and so on). Table 1 includes a selection of these pipelines and Table 2 provides extensive practical guidance for their use in clinical studies. These pipelines address multiple factors that should be given careful consideration when attempting to predict neoantigens for effective cancer treatments. These considerations include: the use of multiple binding prediction algorithms (variability among binding predictions); the integration of both DNA and RNA data (expression of neoantigen candidate genes or transcripts and expression of variant alleles); the phasing of variants (proximal variants detected on the same allele will influence neoantigen sequences) [32, 117]; the interpretation of variants in the context of clonality or heterogeneity [118]; the HLA expression and somatic mutations of patient tumors; and the prediction of tumor immunogenicity [119, 120]. These pipelines are able to provide a comprehensive summary of critical information for each neoantigen prediction, including: variant identity (genomic coordinates, ClinGen allele registry ID, and Human Genome Variation Society (HGVS) variant name); predicted consequence of the variant on the amino acid sequence; corresponding gene and transcript identifiers; peptide sequence; position of the variant within the candidate neoantigen peptide; binding affinity predictions for mutant peptides and the corresponding wild-type peptide sequences; agretopicity value (mutant versus wild-type peptide binding affinity) [121]; DNA variant allele frequency (VAF); RNA VAF; and gene expression values for the gene harboring the variant. Additional data on whether peptides are generated from oncogenic genes, peptide stability, peptide processing and cleavage, and peptide manufacturability should also be considered for final assessment of neoantigens (Table 2).

Several pipelines attempt to integrate DNA and RNA sequencing data by evaluating the VAFs and the gene or transcript expression values of the mutations. Most pipelines currently take into account SNVs and indels, with only a subset considering gene fusion events [8, 32, 122]. Consistent use of the same build or assembly of the genome throughout analysis pipelines, as well as an emphasis on quality control (QC) when performing variant detection and expression analysis, is important for ensuring high confidence in the variants that are detected (Table 2). Once the mutations are confirmed to exist and be expressed, the pipelines then generate a list of neoantigen candidates and consider the probability of cleavage, the location of cleavage, and the TAP transport efficiency of each candidate [8, 123, 124]. The binding affinities of the peptides to the patient-specific MHC molecules are subsequently predicted by using one or more algorithms (Table 1). However, binding-affinity predictions that are made by multiple prediction algorithms vary, and best practices for determining a consensus are poorly articulated at this time. Furthermore, the gold-standard independent validation datasets that exist to evaluate the accuracy of divergent predictions are limited. It remains to be determined whether combining multiple prediction algorithms increases the true positive rate of neoantigen predictions. Some pipelines also consider: (i) manufacturability by measuring peptide characteristics [9]; (ii) immunogenicity by comparing either self-antigens defined by the reference or by the wild-type proteome or known epitopes from viruses and bacteria provided by IEDB [119]; and (iii) pMHC stability [8, 107].

Pipelines vary in their choices of how to rank neoantigens and which specific type of algorithm to use when performing such calculations. Thus, a major challenge lies in how each component should be weighted to create an overall ranking of neoantigens in terms of their potential effectiveness. Kim et al. [125] have attempted to capture the contributions of nine immunogenicity features through the training of machine-learning-based classifiers. Nevertheless, high-quality and experimentally validated neoantigens for training such models remain extremely sparse. In other words, there is no consensus on the features of a ‘good’ neoantigen that would be capable of inducing T cell responses in patients. Furthermore, clinicians may need to consider customized filtering and ranking criteria for individual patient cases, tumor types, or clinical trial designs, details that are not well supported by the existing pipelines. For these reasons, clinical trial efforts should establish an interdisciplinary team of experts analogous to a molecular tumor board for formal quantitative and qualitative review of each patient’s neoantigens. Pipelines such as pVACtools and Vaxrank are designed to support such groups, but there are many important areas in current pipelines that could be improved upon, including: i) consideration of whether the mutation is located within anchor residues for each HLA allele; ii) somatic mutation and expression of patient-specific HLA alleles; iii) the expression level of important cofactors such as genes that are involved in processing, binding, and presentation; and iv) additional factors that influence the manufacturing and delivery of the predicted neoantigens***.***

## Peptide creation, delivery mechanisms, and related analysis considerations for vaccine design

Once neoantigen prioritization is complete, personalized vaccines are designed from predicted immunogenic candidate sequences. Multiple delivery mechanisms exist for use in clinical trials; these include synthetic peptides, DNA, mRNA, viral vectors, and ex-vivo-loaded dendritic cell vaccines [126, 127]. Cancer vaccine delivery is an extensive topic beyond the scope of this review, but other reviews discuss this topic in detail [126–128]. Once a mechanism is chosen and the vaccine is delivered to the patient, professional APCs endocytose the neoantigen sequences. Then, they are processed to generate class-I- and II-restricted MHC peptides for presentation and T cell activation. To design a successful delivery vector, additional analysis steps are necessary to assess peptide manufacturability and to avoid potential incidental DNA vector junctional epitope sequences, or junctions spanning neoantigen sequences that create unintended immunogenic epitopes [8, 129].

Synthetic long peptides (SLPs) are an effective neoantigen delivery mechanism in personalized immunotherapy preclinical studies and clinical trials [30, 101, 130, 131]. These peptides are created from sequences of 15–30 amino acids that contain a core predicted neoantigen. SLPs have greater efficacy than short synthetic peptides, of 8–11 amino acids, because longer peptides require internalization and processing by professional APCs, whereas short peptides can induce immunological tolerance by binding directly to MHC-I on non-professional APCs [132–134]. One limitation of SLPs is manufacturability. Certain chemical properties of the amino acid sequence can make peptides difficult to synthesize, and longer peptides can encounter solubility problems (i.e., they become insoluble). Vaxrank [9] aims to address these concerns by incorporating a manufacturability prediction step in the neoantigen prioritization pipeline. This step measures nine properties that contribute to manufacturing difficulty, including the presence of hydrophobic sequences, cysteine residues, and asparagine-proline bonds. The algorithm then uses this information to choose an ideal window surrounding the somatic mutation for optimum synthesis.

DNA vectors have also delivered neoantigens successfully in a recent preclinical study [135], and DNA neoantigen vaccine clinical trials are currently ongoing in pancreatic and triple-negative breast cancer [136]. Neoantigen encoding DNA sequences can be either directly injected via plasmid vectors using electroporation or incorporated into viral vectors for delivery into patient cells. Adenovirus and vaccinia are the most common viral vectors for personalized vaccines; both are double-stranded DNA (dsDNA) viruses that can incorporate foreign DNA [137]. To maximize neoantigen effectiveness for both vectors, researchers must design sequences with effective junctions and/or spacers. This ensures correct cleavage of the combined sequence by the proteasome as well as the avoidance of inadvertent immunogenic junction antigens. Multiple methods exist to address these challenges.

Furin is a peptidase in the trans-Golgi network that cleaves immature proteins at sequence-specific motifs [138]. Recently, furin-sensitive cleavage sequences were incorporated into a neoantigen DNA vaccine to cleave the sequence into functional neoantigens [135]. EpiToolKit [123] addresses incorrect peptide cleavage in its pipeline by incorporating NetChop [89]. This tool predicts the proteasomal cleavage sites for each neoantigen and can be used to exclude candidates that would undergo inappropriate cleavage. pVACvector, an algorithm included in pVACtools [8], optimizes neoantigen sequence order by running pVACseq on the junction sequences and prioritizing those with low immunogenicity. If high junction immunogenicity cannot be avoided, spacer sequences are included to decrease the potential for inadvertent neoantigens. Taking such analytical considerations into account during personalized vaccine design ensures maximum treatment efficacy in patients.

## T cell recognition, TCR profiling, and immune cell profiling to evaluate response

The ultimate objective of introducing a neoantigen-derived vaccine is to elicit and/or expand a tumor-specific T cell response. This can be evaluated by experimental methods that measure T cell activation and activity, or by computational methods that characterize the patient’s TCR repertoire prior to and after immunotherapy. Standard methods such as IFN-γ ELISPOT assays [139] or MHC multimer assays [140] are beyond the scope of this review, but have been used widely for neoantigen validation purposes [28, 141]. T cells individually undergo complex combinatorial rearrangements in the T cell receptor gene loci in order to create unique clonotypes that are responsible for recognizing antigens. This process occurs within the V(D) J region of the gene, particularly the complementarity-determining region 3 (CDR3), which encodes a region of the TCR that is important for recognizing the pMHC complex. Thus, attempts to characterize the TCR repertoire focus on the identification and characterization of CDR3 sequences, which are representative of the unique T cell clones. This process, termed TCR clonotyping, has been used to identify clonal T cell responses to neoantigens following vaccination with a personalized cancer vaccine or after checkpoint blockade therapy [28]. Researchers have also established an association between the size and diversity of a patient’s TCR repertoire and their response to cancer immunotherapies [142]. Changes in the clonality and diversity of the TCR repertoire, observed from either peripheral blood or tumor-infiltrating lymphocytes (TIL), suggest that an antitumor T cell response is occurring, but they are global metrics that do not successfully identify the T cell clonotypes responsible for tumor rejection.

A variety of available technologies and tools allow sequencing and subsequent analysis of the TCR repertoire. Commercial services such as Adaptive, ClonTech, and iRepertoire differ in a number of aspects, including the required starting material, their library preparation methods, the targeted TCR chains and/or CDR regions for sequencing, the supported organisms, and the sequencing platforms used [143]. Several tools exist to identify TCR CDR3 sequences using various types of data, such as output data from focused assays (e.g., Adaptive, ClonTech or CapTCR), bulk tumor RNA-seq [144], and single cell RNA-seq [144, 145], particularly from the TCR alpha and beta genes (*TRA, TRB*). Challenges associated with TCR profiling include the diversity of the repertoire itself, correctly determining the pairing of *TRA* and *TRB* clonotypes, and the subsequent analysis or validation necessary to pair T cell clones with their target neoantigens. Studies have quantified or predicted the T cell richness, or total number of T cell clones, in the peripheral blood of a healthy individual as up to 10^19^ cells [146]. Thus, there is a sampling bias—based upon the blood draw that was taken, the sample used for sequencing, and the input material for library preparation—that prevents complete evaluation of the global T cell repertoire.

TCR profiling requires the alignment of sequencing reads to the reference TCR genes and the assembly of the rearranged clonotypes. MixCR has been used for TCR alignment and assembly in both bulk and single-cell methods [144, 147]. MIGEC [148] is utilized for methods involving the use of unique molecular identifiers, whereas TraCeR is designed specifically for single-cell methods [145]. MiXCR recovers TCR sequences from raw data through alignment and subsequent clustering, which allows the grouping of identical sequences into clonotypes. If sequences are generated from bulk material (e.g., whole blood or bulk TIL), *TRA* and *TRB* sequences cannot be paired to define the T cell clonotypes specifically. They may be inferred on the basis of frequency, but due to the very high diversity of the T cell repertoire, there are often many clonotypes at similar or low frequencies that make deconvolution of *TRA–TRB* pairs difficult. With the advent of single-cell sequencing data, tools such as TraCeR are now able to identify paired alpha–beta sequences within individual cells that have the same receptor sequences and thus have been derived from the same clonally expanded cells [145].

The identification of clonally expanded neoantigen-specific TCRs complements neoantigen prediction and characterization by indicating whether an active T cell response has been stimulated by an immunotherapeutic intervention. Lu et al. [149] recently developed a single cell RNA-seq approach that identifies neoantigen-specific TCRs by culturing TILs with tandem minigene (TMG)-transfected or peptide-pulsed autologous APCs. Experimental validation data for individual neoantigens can then be utilized to train and improve current neoantigen prioritization strategies.

The clonality of the TCR repertoire can be further evaluated to identify T cell clones that may recognize the same neoantigen. Studies have identified oligoclonal T cell populations that converge, with consistent CDR3 motif sequences, to recognize the same neoantigen [150]. Taking into account the diversity of the repertoire, these findings suggest that oligoclonal events are more likely than monoclonal events, and that there is not likely to be one-to-one mapping between T cell clones and neoantigens. Oligoclonal events and the convergence of the T cell repertoire can be better studied with tools such as GLIPH, which was developed to identify consistent CDR3 motifs across [151] T cells in bulk TCR sequencing.

Antitumor T cell responses have been correlated with changes in the infiltrating immune microenvironment. Methods such as CIBERSORT have been developed to characterize cell compositions on the basis of gene expression profiles from tumor samples [152]. Association between immune cell infiltrates and various factors, including somatic mutation, copy number variation, and gene expression, can be explored interactively through TIMER [153]. This topic has been reviewed in more depth elsewhere [154]. A larger selection of available tools related to T cell and immune cell profiling are listed in Table 1. Overall, few studies have focused on the integration of T cell profiling with neoantigen detection, with the exception of that reported in Li et al. [155], in which TCR clones that were identified from RNAseq samples across Cancer Genome Atlas samples were compared to the mutational profiles of tumors, successfully identifying several public neoantigens that are shared across individuals. Owing to the limited availability of peripheral blood samples and TCR sequencing data with matched tumor DNA or RNA sequencing, one major area for development in the field remains the aggregation of these data and the introduction of an appropriate supervised approach to identify TCR–neoantigen pairs. Such progress would leverage the available data to enhance the identification of neoantigens and to optimize personalized medicine approaches for cancer immunotherapy.

## Conclusions and future directions

Great strides have been made in developing pipelines for neoantigen identification, but there is significant room for improvement. Tools for the rational integration of the myriad complex factors described above are needed. In some cases, useful tools exist but have not been incorporated into analysis workflows. In other cases, factors we believe are important are not being considered because of a lack of tools.

Variant types beyond SNVs and indels have been confirmed as neoantigen sources, but there remains little support for them in current pipelines. Fusions have recently been incorporated into pipelines such as pVACfuse (a tool within pVACtools [8]), INTEGRATE-neo [32], and NeoepitopePred [122]. However, additional genomic variant types that lead to alternative isoforms and to the expression of normally non-coding genomic regions remain unsupported, despite preliminary analyses suggesting their importance. An additional orthogonal, but poorly supported, neoantigen source is the proteasome, which was found to be capable of creating novel antigens by splicing peptides from diverse proteins to create a single antigen [156]. Several computational tools exist to predict post-translational modifications and alternative translation events from sequencing data, such as GPS [157] and KinasePhos [158] for phosphorylation events and altORFev [159] for alternative ORFs. To determine the immunogenicity of these alternative peptides, any tumor-specific predicted sequences could be input into neoantigen prediction software.

The low accuracy of class II HLA typing algorithms has impeded extensive class II neoantigen prediction. When clinical class II HLA typing data are available, they should be used in place of computational HLA predictions in pipelines to improve prediction reliability. In addition, although somatic alterations in HLA gene loci and in the antigen presentation machinery have been implicated in immunotherapeutic resistance, these properties have not been leveraged in predicting neoantigen candidates. HLA gene expression is more often summarized at the gene rather than the allele level. Furthermore, HLA expression is commonly determined from bulk tumor RNAseq data, which are derived from normal, stromal, and infiltrating immune cells, all of which may express HLA genes. The relationship between the present HLA alleles and a predicted neoantigen profile has not been studied, and it remains to be seen whether neoantigens that are restricted to absent or mutant HLA alleles should be specifically filtered out.

For the neoantigen prediction step, mutation positions in the neoantigen should be carefully considered if they occur in anchor residues, since the core sequence of these peptides would be unaffected and identical to that of the wild-type protein. There is also a bias towards class I neoantigen prediction because there are fewer binding-affinity training data and fewer algorithms for class II neoantigens because of their increased MHC binding complexity. Studies have also shown low consensus across MHC binding predictors [8]. pVACtools [8] addresses this challenge by running multiple algorithms simultaneously and reporting the lowest or median score, but a more definitive method for obtaining a binding-affinity consensus remains to be developed. Neoantigen prediction pipelines could also benefit from the inclusion of information on the proposed delivery mechanism to improve prioritization and to streamline vaccine creation.

Although TCR sequences have been recognized to be highly polymorphic, TCRs from T cells that recognize the same pMHC epitope may share conserved sequence features. Researchers have started to quantify these predictive features with the hope of modeling epitope–TCR specificity [160]. Multiple tools (such as TCRex, NetTCR, Repitope) now attempt to predict epitope–TCR binding when given specific TCR sequences. By taking into account the binding specificity of the patient’s existing TCR sequences, neoantigen candidates can be further prioritized according to their immunogenicity. A major advance in optimizing treatment strategies may require the integration of pipelines that perform all of the steps necessary for the generation and processing of neoantigens and for the identification of T cell clones that efficiently recognize them.

Implementing a set of best practices to predict high-quality immunogenic neoantigens can lead to improved personalized patient care in the clinic. Predicting and prioritizing neoantigens is, however, a complicated process that involves many computational steps, each with individualized, adjustable parameters (we provide a specific end-to-end workflow based on our current practices at https://pmbio.org/). Given this complexity, the review of candidates by an immunogenomics tumor board with diverse expertise is highly recommended. We have outlined each step in the neoantigen workflow with human clinical trials in mind, but further research is needed in model organisms to facilitate the development of immunotherapies for human use. Improving neoantigen characterization tools to support the in silico modeling of immune response, model organism systems, human derived samples, and human patient trials is an essential step for improving patient response rates across cancer types.

## Abbreviations

APC: Antigen presenting cell
CDR3: Complementarity-determining region 3
FFPE: Formalin-fixed paraffin-embedded
HLA: Human leukocyte antigen
ICB: Immune checkpoint blockade
IEDB: Immune Epitope Database
Indel: Insertion and deletion
MHC: Major histocompatibility complex
MS: Mass spectrometry
MSI-H: Microsatellite instability-high
NGS: Next generation sequencing
ORF: Open reading frame
pMHC: Peptide-loaded MHC
QC: Quality control
RNA-seq: RNA sequencing
SNV: Single nucleotide variant
SLP: Synthetic long peptides
TCR: T cell receptor
TAP: Transporter associated with antigen processing
TIL: Tumor-infiltrating lymphocytes
VAF: Variant allele frequency
WES: Whole exome sequencing
WGS: Whole genome sequencing
